# Cross-platform evaluation of LLM-generated educational texts on cardiac myxoma: quality, readability, and actionability using network analysis and latent profile analysis

**DOI:** 10.3389/fcvm.2026.1828810

**Published:** 2026-06-10

**Authors:** Bo Deng, Zhiqiang Wang, Tong Cheng, Zhiwen Zhang, Muwei Li

**Affiliations:** 1 Department of Oncology, The First Affiliated Hospital of Hubei University of Science and Technology/Xianning Central Hospital, Xianning, Hubei, China; 2 Yangtze University Medical School, Jingzhou, Hubei, China; 3 Department of Cardiology, Fuwai Central China Cardiovascular Hospital (Central China Fuwai Hospital of Zhengzhou University), Zhengzhou, Henan, China

**Keywords:** actionability, cardiac myxoma, generative artificial intelligence, large language models, latent profile analysis, network analysis, patient education, readability

## Abstract

**Background:**

Patients with cardiac myxoma require long-term follow-up, and the quality of patient education as well as the ability to recognize early symptoms may influence integrated postoperative cardiovascular and tumor-related management. Although large language models (LLMs) have been increasingly applied in medical education, cross-platform empirical evidence in this intersecting context remains limited. This study evaluated the performance and limitations of widely used LLMs in generating patient education texts for cardiac myxoma using a standardized, expert-curated educational question set.

**Methods:**

We constructed a standardized dataset of 60 expert-curated educational questions spanning the disease course of cardiac myxoma and used nine widely used LLMs to generate 540 patient education texts. Text quality, readability, and actionability were assessed using the Patient Education Materials Assessment Tool for Printable Materials (PEMAT-P), Ensuring Quality Information for Patients (EQIP-36), the Global Quality Score (GQS), and seven readability formulas. In addition, a regularized Gaussian graphical model-based partial correlation network analysis and latent profile analysis were performed to identify relationships among evaluation metrics and cross-platform text phenotypes.

**Results:**

Texts generated across platforms showed significant heterogeneity in information quality, understandability, and objective readability, whereas clinical actionability was generally low. Word count showed the strongest positive correlation with the total EQIP-36 score and occupied a central position in the network. Reading-difficulty indices were consistently negatively correlated with PEMAT-P actionability. Latent profile analysis identified three text phenotypes: moderate-quality/low-readability, high-quality/high-actionability, and low-quality/easy-to-read. Ideally suited patient education texts accounted for only a very small proportion of all outputs.

**Conclusions:**

Within this expert-curated educational question set, the current application of LLMs in patient education for cardiac myxoma is primarily limited by reading burden and insufficient behavioral guidance. Although longer outputs were generally associated with higher informational quality scores, greater syntactic complexity was associated with lower actionability of the materials. In addition, latent profile analysis suggested that only a small subset of outputs approached a more favorable quality–actionability profile.

## Introduction

Cardiac myxoma is the most common primary cardiac tumor and arises most frequently in the left atrium (1–3). Although histologically benign, cardiac myxoma carries a substantial risk of functionally malignant clinical consequences because of its friable nature and propensity to fragment and embolize with the bloodstream, potentially resulting in fatal systemic embolism or acute intracardiac obstruction (4, 5). Surgical resection remains the standard curative treatment; however, patients may still face long-term risks of local recurrence or residual microembolic burden after surgery (6, 7). Effective postoperative surveillance and long-term cardiovascular management depend heavily on patients' ability to recognize complications and prodromal symptoms at an early stage (8). Because cardiac myxoma is rare and its pathophysiology lies at the intersection of oncology and acute cardiovascular care, conventional patient education materials often impose substantial cognitive demands, which may delay patients' responses to sudden stroke or heart failure.

The deployment of large language models (LLMs) is reshaping how patients access multidisciplinary medical information (9–11). Generative artificial intelligence (AI) models have been increasingly used for self-education in patients with complex neoplastic diseases and chronic conditions (11–13). To determine the clinical applicability of AI-generated medical texts, it is necessary to evaluate content quality, reading difficulty, and the ability of the material to support patient adherence and action (11, 14, 15). Existing studies have generally characterized text profiles using basic variables such as word count and sentence count; assessed content accuracy and clinical reliability using the Global Quality Score (GQS) and the Ensuring Quality Information for Patients instrument (EQIP-36); quantified linguistic readability with seven readability formulas, including the Automated Readability Index (ARI), Coleman-Liau Index (CLI), Flesch-Kincaid Grade Level (FKGL), Flesch Reading Ease Score (FRES), Gunning Fog Index (GFOG), Linsear Write Formula, and Simple Measure of Gobbledygook (SMOG); and evaluated understandability and actionability using the Patient Education Materials Assessment Tool for Printable Materials (PEMAT-P), including its understandability and actionability domains (11, 16). However, current evaluations of model performance have focused primarily on common solid tumors or single cardiovascular-metabolic conditions, and cross-platform empirical comparisons in the specific clinical context of cardiac myxoma remain lacking.

Previous studies evaluating medical texts have relied predominantly on descriptive statistics based on single dimensions and therefore have been unable to elucidate the complex interactions among different quality indicators (17). Network analysis provides a topological framework for quantifying node centrality and edge weights among multiple text evaluation metrics (18). Latent profile analysis (LPA), as a person-centered statistical approach, can effectively identify unobserved heterogeneous subgroup structures in multidimensional data (19, 20). Therefore, this study used the above evaluation tools and quantitative indices to comprehensively assess the text performance of nine large language models—Ant Afu, ChatGPT 5.2-Thinking, DeepSeek-V3.2, Doubao, ERNIE Bot 5.0 Preview, Gemini 3-pro, KIMI-K2.5 Thinking, Qwen3-Max-Thinking-Preview, and Tencent Yuanbao—in generating responses to a standardized, expert-curated educational question set on cardiac myxoma. By introducing network analysis, we explored the topological relationships among content quality, readability, and actionability metrics; by applying LPA, we further identified latent performance clusters of AI-generated texts across platforms. Rather than directly eliciting real-world patient information needs, this study aimed to provide a reproducible cross-platform benchmark for comparing the textual quality, readability, and actionability of LLM-generated educational responses across the disease course of cardiac myxoma.

## Methods

### Study design and question collection

This study aimed to construct a standardized, clinically relevant educational question set on cardiac myxoma for cross-platform evaluation of large language model (LLM)-generated texts. It did not directly elicit real-world patient information needs from patients, caregivers, search behavior, surveys, or qualitative interviews. Instead, the goal was to develop a reproducible set of educational prompts spanning the disease course of cardiac myxoma, thereby providing a basis for comparing the quality, readability, and actionability of LLM-generated texts in a standardized manner. To this end, we conducted a systematic review of relevant literature and authoritative clinical guidelines and, in combination with expert discussion, preliminarily developed a question bank comprising 60 educational questions (Table 1). According to the knowledge framework of whole-course management for cardiac myxoma, the question bank was categorized into six thematic modules: (1) basic concepts and epidemiology; (2) clinical manifestations and complications; (3) diagnostic pathways and differential diagnosis; (4) treatment strategies and perioperative management; (5) etiology, risk factors, and genetic-related issues; and (6) prognosis, recurrence, follow-up, and lifestyle management. This development process was informed by previous similar studies to ensure the comprehensiveness of topic coverage and the clinical applicability of the questions (11, 21, 22).

**Table 1:** Standardized expert-curated educational questions on cardiac myxoma.

Issue list
Foundational Concepts and Epidemiology
1. What is a cardiac myxoma?
2. Is cardiac myxoma a primary cardiac tumor?
3. Is cardiac myxoma common among primary cardiac tumors?
4. Is cardiac myxoma generally considered a benign tumor?
5. Does cardiac myxoma most often occur in the left atrium (LA)?
6. Does cardiac myxoma most often arise from the interatrial septum at or near the fossa ovalis?
7. What is the typical age at onset of cardiac myxoma?
8. Are there sex-related differences in the epidemiology of cardiac myxoma?
9. Can cardiac myxoma be completely asymptomatic?
10. Can cardiac myxoma trigger atrial fibrillation (AF)?
Etiology, Risk Factors, and Genetics
1. Is the etiology of cardiac myxoma well defined?
2. Which cell type(s) or tissue(s) are implicated as the origin of cardiac myxoma?
3. Is cardiac myxoma classified into sporadic and familial/syndrome-associated forms?
4. What is Carney complex (CNC)?
5. Does Carney complex (CNC) substantially increase the risk of cardiac myxoma?
6. When Carney complex (CNC) is suspected, should genetic testing (e.g., PRKAR1A) be considered?
7. Should first-degree relatives of patients with cardiac myxoma be screened?
8. Can lifestyle interventions (e.g., diet and exercise) prevent cardiac myxoma?
9. Is there a definitive causal relationship between cardiac myxoma and infectious etiologies?
10. Which factors are associated with a higher risk of recurrence?
Clinical Manifestations and Complications
1. Can cardiac myxoma cause exertional dyspnea?
2. Can cardiac myxoma cause orthopnea or paroxysmal nocturnal dyspnea?
3. Can left atrial myxoma mimic mitral stenosis?
4. Can symptoms fluctuate with changes in body position?
5. Can cardiac myxoma cause syncope?
6. Can cardiac myxoma cause stroke?
7. Can cardiac myxoma cause peripheral arterial embolism?
8. Can right-sided cardiac myxoma cause pulmonary embolism?
9. Can cardiac myxoma cause fever?
10. Is cardiac myxoma associated with sudden death?
Diagnostic Pathway and Differential Diagnosis
1. When cardiac myxoma is suspected, is transthoracic echocardiography (TTE) the preferred initial test?
2. What is the primary value of transthoracic echocardiography (TTE) for diagnosing myxoma?
3. What are the limitations of transthoracic echocardiography (TTE) in assessing left atrial myxoma?
4. When is transesophageal echocardiography (TEE) indicated?
5. Can cardiac magnetic resonance (CMR) help distinguish tumor vs. thrombus?
6. What is the primary role of cardiac computed tomography (CT) in evaluating myxoma?
7. In assessing a cardiac mass, what does positron emission tomography/computed tomography (PET/CT) primarily help clarify?
8. Is biopsy typically required to confirm cardiac myxoma?
9. How can left atrial myxoma be differentiated from left atrial thrombus?
10. How can cardiac myxoma be differentiated from infective endocarditis vegetations?
Treatment Strategy and Perioperative Management
1. After diagnosis, is surgical resection generally recommended for cardiac myxoma?
2. What is the key rationale for expediting surgery once myxoma is diagnosed?
3. Is routine preoperative assessment of cardiac function required?
4. Is preoperative coronary evaluation necessary?
5. Can myxoma resection be performed using minimally invasive or robotic approaches?
6. Does complete excision of the tumor stalk and attachment site with an adequate margin reduce recurrence?
7. Can preoperative anticoagulation reduce embolic risk?
8. After ischemic stroke, should the timing of myxoma surgery be reassessed?
9. What are the most common perioperative complications of myxoma resection?
10. Are radiotherapy or chemotherapy routinely indicated for cardiac myxoma?
Prognosis, Recurrence, Follow-up, and Lifestyle Management
1. Is prognosis after surgical resection of cardiac myxoma generally favorable?
2. Can sporadic cardiac myxoma recur after surgery?
3. Is recurrence risk higher in Carney complex (CNC)–associated myxoma?
4. Is echocardiography the cornerstone of postoperative surveillance?
5. Does echocardiographic surveillance typically need to be long term?
6. Which symptom most strongly suggests recurrence and warrants prompt reassessment?
7. How soon after surgery can patients typically resume usual physical activity?
8. Is high-intensity exercise training appropriate after surgery?
9. Does cardiac myxoma diagnosed during pregnancy require special management?
10. When Carney complex (CNC) is suspected or confirmed, is lifelong follow-up required?

To ensure content validity and standardized wording, the question list was independently reviewed by three cardiovascular experts with more than 10 years of clinical experience. The review focused on the alignment between individual questions and their thematic modules, clinical accuracy, suitability for patient education, and the appropriateness of risk-related wording. Items with disagreement were revised through consensus-based discussion, and the final dataset was then established. The resulting expert-curated question set was used as a standardized input framework for cross-platform text generation and evaluation. Accordingly, this study should be interpreted as a comparative assessment of LLM-generated educational texts based on a disease-course-spanning expert-defined prompt set, rather than as a direct assessment of actual patient-derived information needs.

### AI platform question answering and data collection

This study adopted a cross-sectional design and included nine representative large language models (LLMs) from domestic and international conversational artificial intelligence (AI) platforms: Ant Afu (Ant Group, December 15, 2025) (23), ChatGPT 5.2-Thinking (OpenAI, December 11, 2025) (24), DeepSeek-V3.2 (DeepSeek, December 1, 2025) (25), Doubao (ByteDance/Volcano Engine, June 11, 2025) (26), ERNIE Bot 5.0 Preview (Baidu, November 13, 2025) (27), KIMI-K2.5 Thinking (Moonshot AI, January 27, 2026) (28), Qwen3-Max-Thinking-Preview (Alibaba Cloud, January 25, 2026) (29), Gemini 3-pro (Google LLC, November 18, 2025) (30), and Tencent Yuanbao (Tencent, May 30, 2024) (31). These platforms were selected to construct a mixed cross-platform comparison framework that included both China-facing conversational AI platforms and internationally used benchmark models. Specifically, Ant Afu, DeepSeek, Doubao, ERNIE Bot, KIMI, Qwen, and Tencent Yuanbao were included because they are representative AI tools developed by Chinese technology companies or commonly available to Chinese-speaking users. ChatGPT and Gemini were included as internationally recognized global AI tools and reference comparators, because they have been widely discussed and evaluated in previous studies of AI-generated medical information. Their inclusion was intended to benchmark model performance against global AI tools, rather than to imply unrestricted accessibility, clinical availability, or routine deployability in mainland China. Because the regional availability and access policies of commercial LLM platforms may change over time, this study did not evaluate platform accessibility, regulatory compliance, or deployability in China as study outcomes.

All prompts were submitted in English to ensure linguistic consistency across responses. All data were collected through the official web interface or official access channel available to the investigators at the time of data collection. For each of the 60 predefined expert-curated questions, the same prompt was submitted once to each of the nine platforms, generating a total of 540 AI-generated patient education texts. Each question was entered in an independent new conversation on each platform. Before every query, the conversation history was cleared, or a new session was initiated whenever the platform allowed this operation, to minimize residual contextual influence, model memory effects, and personalized interaction history. No follow-up prompts, clarifying questions, prompt optimization, response regeneration, translation, or manual content editing were performed after the initial model response.

**Figure 1:**
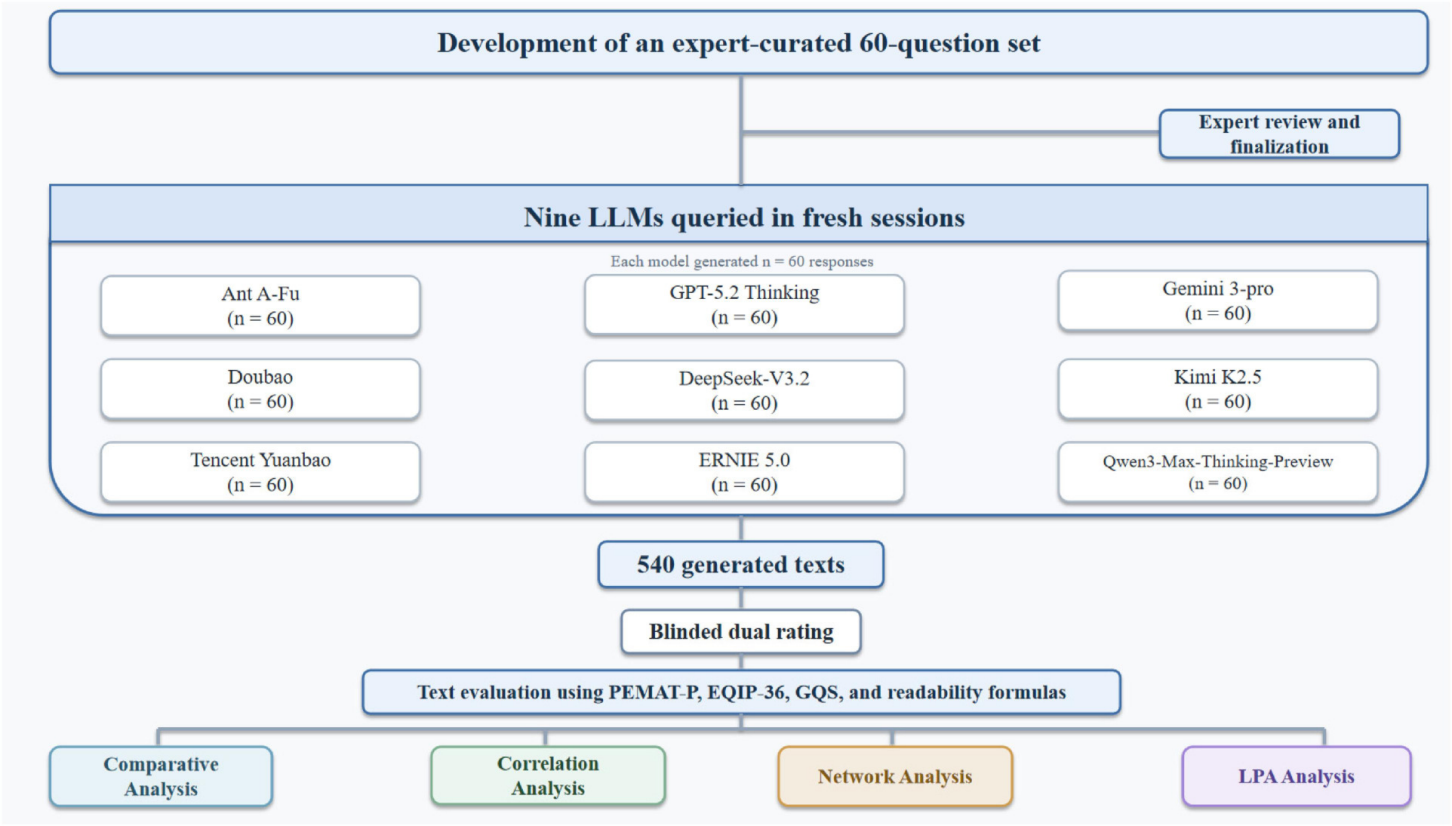
Workflow of expert-curated question-set development, cross-platform LLM querying, blinded evaluation, and downstream analyses.

On February 25, 2026, all questions were entered into each platform at a unified time point by trained investigators using the same standardized procedure. For every interaction, the platform name, model/version displayed at the time of querying, question category, original prompt, generated response, word count, and sentence count were recorded in a structured data extraction file. All AI-generated responses were copied and archived immediately after generation and were preserved verbatim without deletion, rewriting, correction, or post-processing before subsequent evaluation and analysis. The use of a fixed, expert-curated question set was intended to maximize cross-platform comparability rather than to reproduce the full heterogeneity of real-world patient questioning behavior (Figure 1).

This workflow illustrates the full study procedure. First, a standardized set of 60 educational questions on cardiac myxoma was developed through literature review, guideline review, and expert discussion. The question set covered six thematic modules across the disease course of cardiac myxoma. Second, each question was independently submitted to nine LLM platforms in English, with a new or cleared conversation used for each query. This process generated 540 AI-generated patient education texts. Third, all responses were preserved verbatim, de-identified, randomly ordered, and assessed by two blinded raters using PEMAT-P, EQIP-36, and GQS. Fourth, objective readability indices were calculated using seven established readability formulas. Finally, cross-platform comparison, thematic-dimension comparison, Spearman correlation analysis, regularized partial correlation network analysis, and latent profile analysis were performed. LLM, large language model; PEMAT-P, Patient Education Materials Assessment Tool for Printable Materials; EQIP-36, Ensuring Quality Information for Patients; GQS, Global Quality Score.

### Evaluation tools and metrics

To comprehensively evaluate the outputs generated by the nine large language models (LLMs)—Ant Afu, ChatGPT 5.2-Thinking, DeepSeek-V3.2, Doubao, ERNIE Bot 5.0 Preview, Gemini 3-pro, KIMI-K2.5 Thinking, Qwen3-Max-Thinking-Preview, and Tencent Yuanbao—this study applied three categories of evaluation tools and metrics to assess different dimensions of medical information quality. These instruments were used to evaluate the understandability, actionability, information quality, and objective readability of the generated texts. Among them, the Patient Education Materials Assessment Tool for Printable Materials (PEMAT-P), the Ensuring Quality Information for Patients instrument (EQIP-36), and the Global Quality Score (GQS) were all scored using a blinded, independent dual-rater approach. These measures were selected to characterize the textual properties of LLM-generated educational materials; they were not designed to directly assess real-world patient comprehension, factual accuracy, hallucination risk, or guideline concordance.

PEMAT-P, EQIP-36, and GQS assessments were independently performed by two raters who received standardized training. Before formal scoring, the raters developed detailed scoring criteria and decision examples based on the original manuals of the instruments, and they conducted pilot testing and calibration using 30 randomly selected texts to establish the final scoring rules. To reduce information bias, all texts were de-identified and randomly ordered before assessment. The raters were blinded to both the platform source and the topic category and were given access only to the text content. Interrater agreement was high in this study (κ = 0.887). If the discrepancy between the two raters on any scale exceeded the predefined threshold, a senior researcher with a cardiovascular background served as a third reviewer and adjudicated the final score through discussion. All other disagreements were resolved by consensus after re-examination of the scoring rationale.

### Understandability and actionability assessment

The Patient Education Materials Assessment Tool for Printable Materials (PEMAT-P) was used to assess the understandability and actionability of patient education materials (32). Higher PEMAT-P scores indicate that a text is easier to understand and more likely to facilitate actionable responses by readers (32).

### Quality assessment

First, the Ensuring Quality Information for Patients instrument (EQIP-36), a 36-item scale for evaluating the quality of patient information, was used. This instrument covers three major domains—content, source identification, and structure—and is designed to assess the comprehensiveness, accuracy, and standardization of health information. The total score reflects the overall level of information quality and is presented as an overall quality score ranging from 0 to 100 (33). The EQIP-36 has been used to evaluate the quality of online health information and has demonstrated good reliability and validity in related studies (34).

Second, the Global Quality Score (GQS), a global rating scale for overall information quality, was applied. Using this instrument, raters subjectively evaluated the overall usefulness and reliability of each response on a 5-point scale, ranging from 1 (very poor quality) to 5 (very high quality) (35).

### Readability metrics

Seven classic English-language readability formulas were used to calculate readability metrics for each response, including the Automated Readability Index (ARI), Coleman-Liau Index (CLI), Flesch-Kincaid Grade Level (FKGL), Flesch Reading Ease Score (FRES), Gunning Fog Index (GFOG), Linsear Write Formula, and Simple Measure of Gobbledygook (SMOG) (36–41). These metrics estimate reading difficulty by analyzing lexical and sentence-level features of the text, and each metric has its own scoring range and interpretation. Among them, six indices other than FRES correspond to US grade levels, with higher scores indicating a higher educational level required for comprehension and greater textual complexity. In contrast, FRES ranges from 0 to 100, with higher scores indicating that the text is easier to understand. All readability metrics were calculated using the dedicated software tool ReadabilityFormulas.com to ensure a standardized assessment process. Readability analysis enabled an objective comparison of the linguistic complexity of responses across platforms and, when interpreted together with subjective ratings, helped determine whether the generated content was appropriate for reading and comprehension by the general public.

### Statistical analysis

Continuous variables were summarized as median and interquartile range, expressed as M (Q1, Q3). Comparisons between two groups were performed using the Mann–Whitney *U*-test, whereas comparisons among multiple groups were conducted using the Kruskal–Wallis *H*-test. When the overall difference was statistically significant, Dunn *post hoc* pairwise comparisons were performed with Bonferroni correction for multiple testing. Correlations were assessed using Spearman rank correlation coefficients. All tests were two-sided, with a significance level of α = 0.05. Because each of the 60 predefined questions was posed to all nine LLM platforms, platform-level comparisons also involved matched observations at the question level. Therefore, in addition to the primary nonparametric analyses, we performed question-matched sensitivity analyses to account for within-question dependence across platforms. Specifically, overall between-platform differences were re-evaluated using the Friedman test, with question treated as the repeated-measures block; when the overall Friedman test was significant, *post hoc* pairwise comparisons were performed using the Wilcoxon signed-rank test with Bonferroni correction. The purpose of these additional analyses was to determine whether the main findings remained robust after accounting for the matched data structure.

Latent profile analysis (LPA) was conducted in Mplus version 8.11, and models with one to five classes were fitted. As the number of classes increased, the log-likelihood continuously improved, whereas the Akaike information criterion (AIC), Bayesian information criterion (BIC), and sample-size adjusted Bayesian information criterion (SABIC) generally decreased. Considering the information criteria, bootstrap likelihood ratio test (BLRT), entropy, and the minimum class proportion, the three-class model was ultimately selected as the optimal solution.

To characterize the conditional dependence structure among understandability, actionability, information quality, overall quality, readability burden, and text length in large language model (LLM)-generated patient education texts on cardiac myxoma, a partial correlation network analysis based on a regularized Gaussian graphical model (GGM) was performed. The network included six continuous indicators: PEMAT-P understandability score, PEMAT-P actionability score, total EQIP-36 score, total Global Quality Score (GQS), SMOG grade level, and word count (Words). Before network estimation, all continuous variables were subjected to a nonparanormal (NPN) transformation to reduce the influence of non-normality on GGM estimation. Subsequently, a regularized partial correlation network was estimated in R version 4.5.2 using the extended Bayesian information criterion graphical least absolute shrinkage and selection operator (EBICglasso) method, with L1 regularization applied to obtain a sparse network structure. The network was presented as a weighted signed graph, in which edges represented the direction and strength of conditional associations after controlling for all other nodes. Model selection was based on the extended Bayesian information criterion (EBIC), with γ = 0.5. Missing data were handled by pairwise deletion; however, no missing values were identified in the present study. The accuracy of edge weights and the stability of centrality indices were evaluated using 2,000 bootstrap resamples, and the consistency of network results across different sampling proportions was examined using the case-dropping procedure. Strength was used as the primary centrality metric for interpretation.

Conventional statistical analyses were performed using IBM SPSS Statistics version 25. LPA was conducted in Mplus version 8.11, network analysis was performed in R version 4.5.2, and visualization was generated using GraphPad Prism version 9 and related network-analysis packages.

## Results

### Text characteristics

#### Characteristics of patient education texts on cardiac myxoma generated by different large language model platforms

A total of 540 patient education texts on cardiac myxoma generated by nine large language model (LLM) platforms were included, with 60 texts from each platform (Tables 2, 3). Kruskal–Wallis tests showed statistically significant between-platform differences in the Patient Education Materials Assessment Tool for Printable Materials (PEMAT-P) understandability, actionability, and composite scores; the 36-item Ensuring Quality Information for Patients instrument (EQIP-36); the Global Quality Score (GQS); text length; and multiple readability indices (all *P* < 0.001). ERNIE Bot 5.0 Preview achieved the highest PEMAT-P understandability score [median, 83.95 (76.43, 84.60)], whereas Qwen3-Max-Thinking-Preview had the lowest [50.00 (41.70, 61.50)]. The highest PEMAT-P composite scores were observed for ERNIE Bot 5.0 Preview and Gemini 3-pro (both 66.70), while Qwen3-Max-Thinking-Preview had the lowest score [41.20 (35.30, 50.00)]. Overall actionability was low (overall median, 20.00); DeepSeek-V3.2 and Gemini 3-pro performed relatively better (both 40.00), whereas Doubao scored the lowest [16.70 (0.00, 20.00)]. For EQIP-36, ERNIE Bot 5.0 Preview and KIMI-K2.5 Thinking yielded the highest scores (45.59 and 45.23, respectively), whereas Qwen3-Max-Thinking-Preview had the lowest score [34.30 (30.89, 36.88)]. For the GQS, the median score was 5 for Doubao, ERNIE Bot 5.0 Preview, and Tencent Yuanbao, and generally 4 for the remaining platforms. ERNIE Bot 5.0 Preview generated the longest texts (median word count, 780; median sentence count, 41), whereas Qwen3-Max-Thinking-Preview generated the shortest (median word count, 164; median sentence count, 9). In terms of readability, ChatGPT 5.2-Thinking-generated texts were relatively more difficult to read [e.g., Automated Readability Index (ARI), 20.34], whereas ERNIE Bot 5.0 Preview-generated texts were comparatively more readable, with a Flesch Reading Ease Score (FRES) of 30.66, an ARI of 15.66, and a Gunning Fog Index (GFOG) of 16.75.Question-matched sensitivity analyses using the Friedman test showed that the overall pattern of between-platform differences remained materially unchanged after accounting for within-question dependence. Significant overall differences remained for the major quality, actionability, and readability outcomes, and *post hoc* Wilcoxon signed-rank comparisons with Bonferroni correction yielded results broadly consistent with the primary analyses (all key outcomes, *P* < 0.05).The corresponding Kruskal–Wallis effect sizes ranged from ε^2^ = 0.040 to 0.353 across the major quality, actionability, and readability outcomes, indicating small-to-moderate to moderate between-platform effects. Larger effects were observed for text-length indicators, whereas actionability-related differences were comparatively smaller.In question-matched sensitivity analyses, Kendall's W values for the major outcomes were generally in the small-to-moderate range, suggesting that the overall pattern of cross-platform differences remained materially robust after accounting for within-question dependence.

**Table 2:** Cross-platform comparison of text quality and actionability metrics in LLM-generated educational texts on cardiac myxoma.

Variables	Total (*n* = 540)	Ant Afu (*n* = 60)	ChatGPT 5.2-thinking (*n* = 60)	DeepSeek-V3.2 (*n* = 60)	Doubao (*n* = 60)	ERNIE bot 5.0 preview (*n* = 60)	Gemini 3-pro (*n* = 60)	KIMI-K2.5 thinking (*n* = 60)	Qwen3-max-thinking-preview (*n* = 60)	Tencent yuanbao (*n* = 60)	*P*
PEMAT-P Understandability, M (Q₁, Q₃)	76.90 (69.20, 84.60)	75.00 (69.20, 76.90)	75.00 (65.93, 81.30)	70.30 (61.50, 76.90)	76.90 (75.00, 83.62)	83.95 (76.43, 84.60)	78.60 (76.90, 84.60)	76.90 (69.20, 84.60)	50.00 (41.70, 61.50)	76.90 (71.05, 84.60)	<.001
PEMAT-P Actionability, M (Q₁, Q₃)	20.00 (0.00, 44.67)	26.65 (0.00, 60.00)	20.00 (0.00, 52.50)	40.00 (19.18, 60.00)	16.70 (0.00, 20.00)	20.00 (20.00, 60.00)	40.00 (10.73, 50.00)	20.00 (20.00, 40.00)	20.00 (0.00, 40.00)	20.00 (0.00, 50.00)	<.001
PEMAT-P score, M (Q₁, Q₃)	61.10 (52.90, 69.70)	61.10 (55.60, 69.25)	59.40 (50.00, 66.70)	58.95 (50.00, 72.20)	60.55 (52.90, 66.70)	66.70 (61.10, 72.20)	66.70 (59.27, 74.18)	61.10 (55.60, 70.60)	41.20 (35.30, 50.00)	61.10 (54.48, 72.33)	<.001
EQIP-36 score, M (Q₁, Q₃)	41.18 (34.29, 48.57)	39.85 (34.17, 44.62)	43.59 (32.22, 51.41)	44.73 (35.04, 48.96)	40.91 (34.84, 48.49)	45.59 (40.96, 51.90)	38.60 (34.29, 48.58)	45.23 (40.88, 51.56)	34.30 (30.89, 36.88)	37.69 (32.90, 50.35)	<.001
GQS score, M (Q₁, Q₃)	4.00 (4.00, 5.00)	4.00 (4.00, 4.00)	4.00 (4.00, 4.00)	4.00 (4.00, 4.00)	5.00 (4.00, 5.00)	5.00 (4.00, 5.00)	4.00 (3.00, 4.00)	4.00 (4.00, 5.00)	4.00 (3.00, 4.00)	5.00 (4.00, 5.00)	<.001

Data are presented as median (Q1, Q3). *P* values were derived from the Kruskal–Wallis test.

PEMAT-P, patient education materials assessment tool for printable materials; EQIP-36, ensuring quality information for patients instrument; GQS, global quality score; Q1, first quartile; Q3, third quartile.

**Table 3:** Cross-platform comparison of text length and readability metrics in LLM-generated educational texts on cardiac myxoma.

Variables	Total (*n* = 540)	Ant Afu (*n* = 60)	ChatGPT 5.2-thinking (*n* = 60)	DeepSeek-V3.2 (*n* = 60)	Doubao (*n* = 60)	ERNIE Bot 5.0 preview (*n* = 60)	Gemini 3-pro (*n* = 60)	KIMI-K2.5 thinking (*n* = 60)	Qwen3-max-thinking-preview (*n* = 60)	Tencent yuanbao (*n* = 60)	*P*
Words, M (Q₁, Q₃)	512.00 (291.75, 718.00)	458.00 (296.50, 819.00)	358.50 (230.75, 533.75)	632.00 (544.00, 710.25)	482.00 (322.75, 639.00)	780.00 (662.25, 896.75)	606.50 (455.00, 815.00)	458.50 (324.25, 527.00)	164.00 (121.50, 187.25)	586.00 (335.50, 800.00)	<.001
Sentences, M (Q₁, Q₃)	26.00 (14.00, 37.00)	26.00 (19.00, 33.50)	14.00 (8.00, 23.25)	31.00 (25.00, 40.25)	26.00 (17.00, 31.50)	41.00 (32.50, 53.00)	35.50 (25.50, 53.50)	22.50 (17.00, 29.00)	9.00 (6.75, 12.00)	25.50 (12.75, 41.00)	<.001
Characters, M (Q₁, Q₃)	3,278.50 (1,770.00, 4,542.25)	3,093.00 (1,953.00, 5,089.50)	2,154.00 (1,319.25, 3,425.50)	4,068.50 (3,521.00, 4,642.50)	3,274.50 (2,082.50, 4,006.75)	4,521.00 (3,895.75, 5,345.25)	3,804.00 (2,843.00, 4,999.50)	2,988.00 (2,017.25, 3,492.00)	997.00 (746.00, 1,211.00)	3,581.50 (2,000.50, 4,986.50)	<.001
Letters, M (Q₁, Q₃)	3,019.00 (1,654.25, 4,186.50)	2,761.00 (1,817.00, 4,648.00)	2,050.50 (1,277.00, 2,919.25)	3,673.00 (3,113.25, 4,171.25)	2,947.00 (1,978.75, 3,769.25)	4,317.00 (3,722.75, 5,130.50)	3,487.50 (2,681.00, 4,573.00)	2,747.50 (1,878.00, 3,280.00)	948.50 (719.75, 1,173.50)	3,380.00 (1,830.50, 4,382.00)	<.001
Syllables, M (Q₁, Q₃)	1,000.50 (538.00, 1,401.00)	940.50 (617.75, 1,475.75)	665.50 (405.00, 943.00)	1,243.50 (1,033.00, 1,400.75)	983.50 (646.50, 1,249.50)	1,444.00 (1,226.25, 1,677.00)	1,165.50 (891.75, 1,558.00)	892.50 (596.00, 1,069.00)	301.50 (240.25, 384.25)	1,075.00 (591.25, 1,422.50)	<.001
Complex Words, M (Q₁, Q₃)	131.00 (69.00, 189.00)	132.50 (86.00, 189.75)	84.00 (44.75, 129.00)	177.50 (141.75, 210.75)	129.00 (86.50, 173.50)	177.00 (148.75, 217.00)	168.50 (111.00, 222.25)	128.50 (67.50, 156.50)	38.00 (26.75, 59.25)	131.50 (67.75, 178.25)	<.001
ARI, M (Q₁, Q₃)	17.85 (15.80, 20.03)	18.11 (15.90, 20.86)	20.34 (18.19, 25.86)	18.88 (17.87, 20.38)	18.28 (16.34, 20.19)	15.66 (14.52, 16.95)	16.81 (15.54, 18.23)	18.34 (15.63, 20.53)	16.73 (14.16, 18.04)	18.41 (16.66, 20.97)	<.001
CLI, M (Q₁, Q₃)	16.61 (15.09, 18.13)	16.93 (15.59, 18.09)	16.16 (14.59, 17.04)	17.09 (15.88, 18.30)	17.73 (15.71, 18.57)	15.75 (14.64, 16.57)	16.37 (14.90, 17.68)	18.32 (16.01, 19.72)	17.57 (15.76, 18.99)	14.96 (13.83, 16.53)	<.001
FKGL, M (Q₁, Q₃)	14.82 (13.12, 16.49)	15.04 (12.94, 16.28)	16.22 (14.09, 18.84)	15.32 (13.70, 16.45)	15.11 (13.64, 16.85)	13.60 (12.54, 14.78)	14.20 (13.11, 15.35)	15.46 (12.91, 17.16)	14.36 (12.10, 16.08)	14.78 (13.33, 16.18)	<.001
FRES, M (Q₁, Q₃)	23.75 (14.85, 33.75)	24.06 (15.59, 32.31)	20.45 (13.34, 32.62)	22.14 (14.29, 26.26)	21.50 (10.23, 31.92)	30.66 (24.46, 36.39)	23.99 (16.78, 30.64)	20.01 (9.41, 33.95)	23.20 (13.79, 35.98)	28.26 (21.42, 38.25)	<.001
GFOG, M (Q₁, Q₃)	18.28 (16.41, 20.05)	18.52 (16.74, 19.54)	19.38 (17.15, 21.52)	19.12 (17.30, 20.13)	19.07 (16.60, 20.43)	16.75 (15.75, 18.94)	17.56 (16.64, 18.68)	18.77 (15.79, 20.58)	17.81 (15.41, 19.92)	18.34 (15.98, 19.77)	<.001
Linsear, M (Q₁, Q₃)	74.00 (69.00, 79.00)	76.00 (73.00, 80.25)	69.00 (65.75, 75.00)	80.00 (75.00, 84.00)	76.00 (69.75, 82.00)	72.00 (68.75, 77.00)	76.00 (72.00, 80.00)	72.00 (67.75, 76.25)	74.00 (68.00, 79.25)	68.50 (65.00, 74.00)	<.001
SMOG, M (Q₁, Q₃)	15.80 (14.46, 17.12)	15.74 (14.54, 16.73)	16.77 (15.23, 18.29)	16.34 (15.30, 17.36)	16.29 (14.81, 17.52)	15.05 (14.29, 16.38)	15.25 (14.20, 16.29)	16.16 (14.11, 17.40)	15.25 (13.70, 16.77)	15.71 (14.19, 17.05)	<.001

Data are presented as median (Q1, Q3). *P* values were derived from the Kruskal–Wallis test.

ARI, automated readability index; CLI, coleman-liau index; FKGL, flesch-kincaid grade level; FRES, flesch reading ease score; GFOG, gunning fog index; SMOG, simple measure of gobbledygook; Q1, first quartile; Q3, third quartile.

#### Characteristics across content dimensions of cardiac myxoma educational texts generated by large language model platforms

The 540 texts were classified into six categories according to content dimension (Tables 4, 5). The Kruskal–Wallis test showed no significant overall differences across dimensions in information quality or in the Patient Education Materials Assessment Tool for Printable Materials (PEMAT-P) metrics: PEMAT-P understandability (*χ*^2^ = 3.19, *P* = 0.67), actionability (*χ*^2^ = 4.20, *P* = 0.52), and total score (*χ*^2^ = 6.18, *P* = 0.289) remained at comparable levels. Similarly, no significant between-dimension difference was observed for the Ensuring Quality Information for Patients instrument (EQIP-36) (*χ*^2^ = 6.67, *P* = 0.246). These findings suggest that the understandability–actionability profile of the generated texts remained relatively stable across topics.

**Table 4:** Comparison of quality and actionability metrics across thematic dimensions in LLM-generated educational texts on cardiac myxoma.

Variables	Total (*n* = 540)	Clinical manifestations and complications (*n* = 90)	Diagnostic pathway and differential diagnosis (*n* = 90)	Etiology, risk factors, and genetics (*n* = 90)	Foundational concepts and epidemiology (*n* = 90)	Prognosis, recurrence, follow-up, and lifestyle management (*n* = 90)	Treatment strategy and perioperative management (*n* = 90)	*P*
PEMAT-P Understandability, M (Q₁, Q₃)	76.90 (69.20, 84.60)	76.90 (67.33, 84.27)	76.90 (66.70, 83.30)	76.90 (69.20, 84.60)	76.90 (69.20, 84.27)	76.90 (67.33, 84.60)	75.00 (62.20, 78.60)	0.67
PEMAT-P Actionability, M (Q₁, Q₃)	20.00 (0.00, 44.67)	20.00 (0.00, 40.00)	20.00 (0.00, 40.00)	33.30 (0.00, 60.00)	20.00 (0.00, 50.00)	20.00 (0.00, 50.00)	20.00 (16.70, 40.00)	0.52
PEMAT-P score, M (Q₁, Q₃)	61.10 (52.90, 69.70)	61.10 (50.00, 66.70)	58.80 (52.68, 66.70)	61.10 (53.57, 72.20)	61.10 (53.72, 71.85)	61.10 (52.68, 72.20)	61.10 (50.00, 66.70)	0.289
EQIP-36 score, M (Q₁, Q₃)	41.18 (34.29, 48.57)	38.62 (33.89, 47.02)	41.12 (35.70, 48.08)	41.29 (34.85, 50.00)	37.32 (31.43, 48.57)	42.98 (35.70, 48.59)	42.86 (34.43, 48.57)	0.246
GQS score, M (Q₁, Q₃)	4.00 (4.00, 5.00)	4.00 (4.00, 5.00)	4.00 (4.00, 5.00)	4.00 (4.00, 5.00)	4.00 (3.00, 4.00)	4.00 (4.00, 5.00)	4.00 (4.00, 5.00)	0.003

Data are presented as median (Q1, Q3). *P* values were derived from the Kruskal–Wallis test.

PEMAT-P, patient education materials assessment tool for printable materials; EQIP-36, ensuring quality information for patients instrument; GQS, global quality score; Q1, first quartile; Q3, third quartile.

**Table 5:** Comparison of text length and readability metrics across thematic dimensions in LLM-generated educational texts on cardiac myxoma.

Variables	Total (*n* = 540)	Clinical manifestations and complications (*n* = 90)	Diagnostic pathway and differential diagnosis (*n* = 90)	Etiology, risk factors, and genetics (*n* = 90)	Foundational concepts and epidemiology (*n* = 90)	Prognosis, recurrence, follow-up, and lifestyle management (*n* = 90)	Treatment strategy and perioperative management (*n* = 90)	*P*
Words, M (Q₁, Q₃)	512.00 (291.75, 718.00)	407.50 (238.00, 642.00)	520.00 (364.00, 667.00)	585.50 (361.00, 716.25)	509.00 (213.50, 712.25)	564.00 (323.50, 814.50)	473.50 (295.50, 737.00)	0.027
Sentences, M (Q₁, Q₃)	26.00 (14.00, 37.00)	22.50 (12.00, 33.50)	26.00 (17.25, 32.75)	30.00 (18.00, 43.00)	30.00 (13.00, 41.00)	24.00 (15.00, 36.75)	22.00 (14.25, 33.75)	0.073
Characters, M (Q₁, Q₃)	3,278.50 (1,770.00, 4,542.25)	2,641.50 (1,463.50, 3,947.00)	3,269.00 (2,228.25, 4,286.50)	3,718.50 (2,251.25, 4,677.00)	3,240.50 (1,412.50, 4,635.75)	3,435.00 (2,030.75, 5,055.25)	2,950.00 (1,859.25, 4,373.25)	0.029
Letters, M (Q₁, Q₃)	3,019.00 (1,654.25, 4,186.50)	2,393.50 (1,333.00, 3,610.50)	2,995.00 (2,111.25, 3,847.00)	3,413.00 (1,965.75, 4,243.00)	2,969.00 (1,316.50, 4,219.00)	3,230.50 (1,933.75, 4,520.75)	2,811.00 (1,683.25, 4,094.50)	0.028
Syllables, M (Q₁, Q₃)	1,000.50 (538.00, 1,401.00)	803.50 (419.00, 1,241.50)	986.00 (700.50, 1,283.50)	1,125.50 (636.25, 1,444.75)	971.50 (442.25, 1,402.75)	1,080.50 (623.00, 1,487.00)	911.50 (560.50, 1,359.00)	0.029
Complex Words, M (Q₁, Q₃)	131.00 (69.00, 189.00)	109.50 (56.75, 163.00)	133.00 (87.00, 178.00)	157.00 (88.00, 200.50)	118.50 (63.25, 198.50)	130.00 (75.25, 197.75)	118.50 (75.00, 177.75)	0.044
ARI, M (Q₁, Q₃)	17.85 (15.80, 20.03)	17.69 (15.54, 19.24)	18.81 (16.08, 20.25)	17.50 (15.91, 19.41)	17.63 (15.36, 19.23)	18.45 (15.56, 21.23)	18.24 (16.17, 20.16)	0.284
CLI, M (Q₁, Q₃)	16.61 (15.09, 18.13)	16.34 (15.20, 18.14)	16.61 (15.45, 17.76)	16.82 (15.29, 18.07)	16.64 (14.60, 18.54)	16.54 (14.84, 17.87)	17.06 (15.00, 18.69)	0.804
FKGL, M (Q₁, Q₃)	14.82 (13.12, 16.49)	14.70 (12.89, 15.90)	15.36 (13.53, 16.66)	14.39 (12.83, 16.46)	14.05 (12.71, 15.61)	15.32 (13.45, 17.12)	14.98 (13.28, 17.05)	0.014
FRES, M (Q₁, Q₃)	23.75 (14.85, 33.75)	24.26 (17.12, 34.51)	23.07 (15.72, 30.48)	22.45 (13.95, 34.26)	25.12 (14.29, 35.51)	24.77 (15.60, 33.79)	23.13 (13.35, 34.07)	0.835
GFOG, M (Q₁, Q₃)	18.28 (16.41, 20.05)	18.25 (16.47, 19.59)	18.95 (17.09, 20.09)	18.11 (16.12, 20.23)	17.56 (15.94, 19.66)	18.75 (16.68, 20.95)	18.23 (16.37, 20.52)	0.073
Linsear, M (Q₁, Q₃)	74.00 (69.00, 79.00)	75.00 (70.00, 80.00)	74.00 (70.00, 78.75)	76.00 (71.00, 80.00)	74.00 (68.00, 80.75)	72.50 (68.00, 77.00)	73.50 (68.00, 79.00)	0.315
SMOG, M (Q₁, Q₃)	15.80 (14.46, 17.12)	15.66 (14.31, 16.74)	16.55 (14.73, 17.25)	15.64 (14.29, 17.25)	15.25 (14.11, 16.53)	16.04 (14.85, 17.75)	16.05 (14.52, 17.30)	0.006

Data are presented as median (Q1, Q3). *P* values were derived from the Kruskal–Wallis test.

ARI, automated readability index; CLI, coleman-liau index; FKGL, flesch-kincaid grade level; FRES, flesch reading ease score; GFOG, gunning fog index; SMOG, simple measure of gobbledygook; Q1, first quartile; Q3, third quartile.

In contrast, text length and lexical complexity varied across dimensions. Texts in the etiology, risk factors, and genetic-related issues dimension were longer and more complex (words, M = 585.50; characters, M = 3,718.50; complex words, M = 157.00), whereas those in the clinical manifestations and complications dimension were the shortest (words, M = 407.50; characters, M = 2,641.50; complex words, M = 109.50). Differences in word count, character count, letter count, syllable count, and number of complex words all reached statistical significance (*P* = 0.027–0.044).

Regarding readability, most indices, including the Automated Readability Index (ARI), Coleman–Liau Index (CLI), Flesch Reading Ease Score (FRES), and Linsear Write Formula, did not differ significantly across dimensions. However, significant between-dimension differences were observed for the Flesch–Kincaid Grade Level (FKGL) and the Simple Measure of Gobbledygook (SMOG) index (*χ*^2^ = 16.19, *P* = 0.006). Specifically, the diagnostic pathways and differential diagnosis dimension and the prognosis, recurrence, follow-up, and lifestyle management dimension showed relatively higher grade-level demands (e.g., SMOG, 16.55 and 16.04, respectively), suggesting that these topics may impose a greater reading burden.

The median Global Quality Score (GQS) was 4 across all dimensions; nevertheless, a statistically significant overall difference was still observed (*χ*^2^ = 17.85, *P* = 0.003).

Effect sizes for thematic-dimension comparisons were generally very small (ε^2^ ≈ 0.001–0.024), supporting the interpretation that most between-dimension differences were limited in practical magnitude.

### Text quality

**Figure 2:**
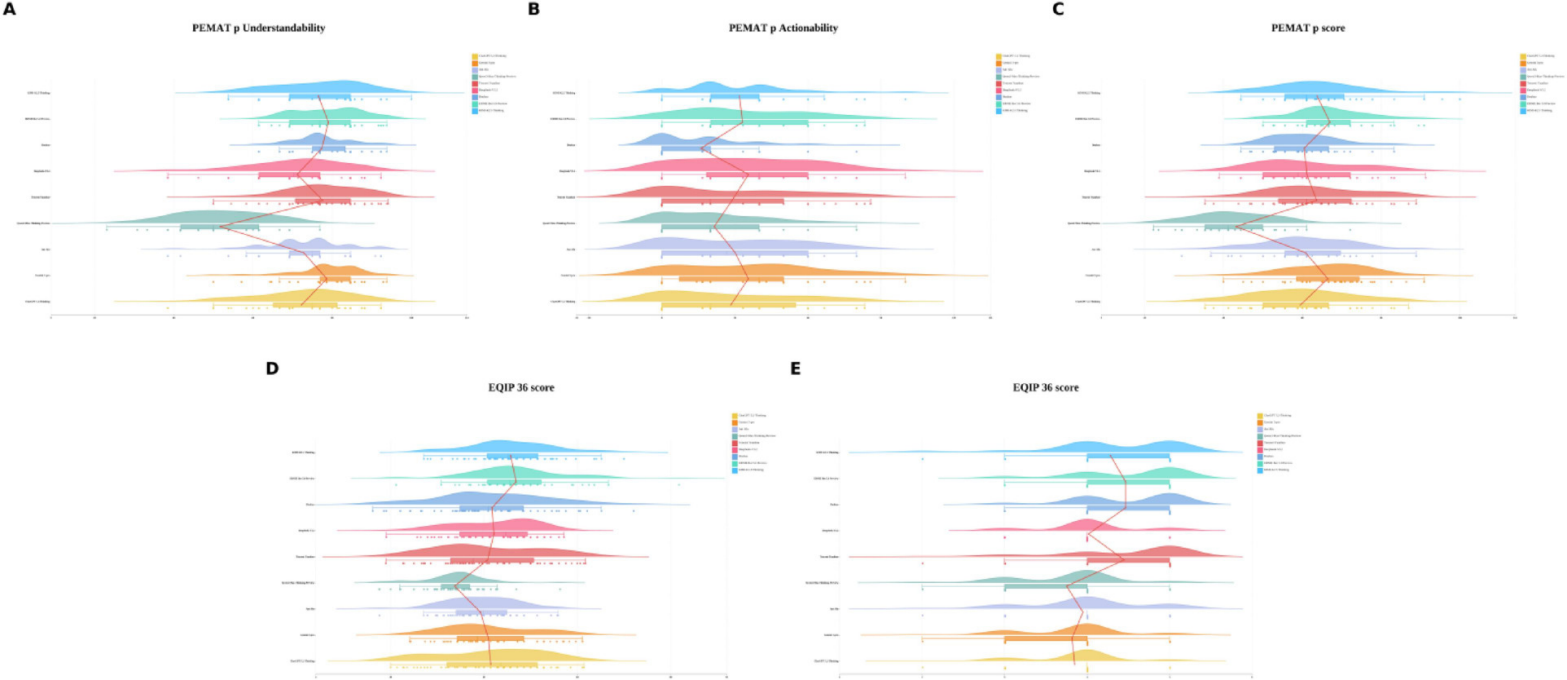
Distributions of text quality, understandability, actionability, and overall quality of cardiac myxoma educational texts generated by different large language model platforms.

As illustrated by the raincloud plots in Figures 2A–C, the distributions of understandability and overall quality scores assessed using the Patient Education Materials Assessment Tool for Printable Materials (PEMAT-P) showed clear stratification across platforms. ERNIE Bot 5.0 Preview and Gemini 3-pro demonstrated an overall rightward shift with higher median scores, indicating clearer information organization and expression. Ant Afu, ChatGPT 5.2-Thinking, Doubao, KIMI-K2.5 Thinking, and Tencent Yuanbao were at an intermediate level. In contrast, Qwen3-Max-Thinking-Preview showed an overall leftward shift accompanied by clustering at lower scores, suggesting relatively unstable and generally poorer quality. Further assessment using the 36-item Ensuring Quality Information for Patients instrument (EQIP-36) in Figures 2D,E likewise showed that ERNIE Bot 5.0 Preview and KIMI-K2.5 Thinking had more right-shifted quality distributions with lower dispersion, whereas Qwen3-Max-Thinking-Preview had the lowest scores and greater variability, underscoring substantial cross-platform differences in text quality.

Raincloud plots show the distribution of quality-related scores for each LLM platform, with 60 generated responses included per platform. Panel A shows PEMAT-P understandability scores, Panel B shows PEMAT-P actionability scores, Panel C shows PEMAT-P composite scores, Panel D shows EQIP-36 scores, and Panel E shows GQS scores. Each dot represents one AI-generated patient education text. The density curve shows the distribution of scores, and the boxplot summarizes the median and interquartile range. Higher PEMAT-P scores indicate better understandability or actionability, higher EQIP-36 scores indicate better information quality, and higher GQS scores indicate better overall quality. PEMAT-P, Patient Education Materials Assessment Tool for Printable Materials; EQIP-36, Ensuring Quality Information for Patients; GQS, Global Quality Score.

### Text readability

**Figure 3:**
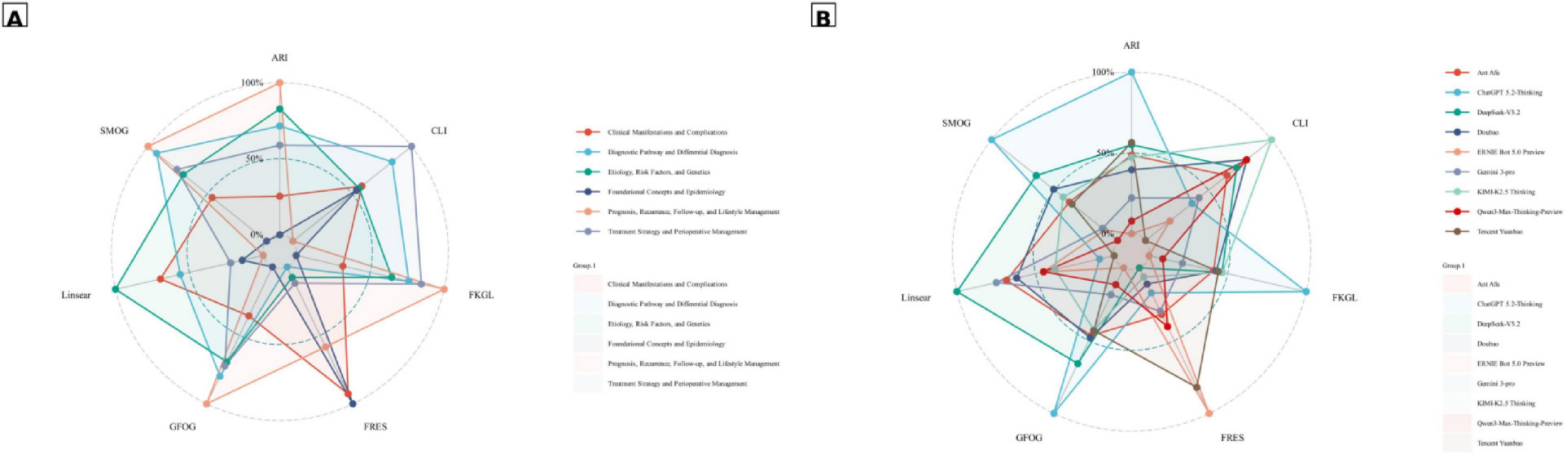
Readability profiles of LLM-generated educational texts on cardiac myxoma across platforms and thematic dimensions.

The radar chart (Figure 3) provides an integrated visualization of the relative levels of seven readability indices. Overall, the readability profiles were broadly similar across thematic dimensions. However, grade-level indices, including the Automated Readability Index (ARI), Flesch-Kincaid Grade Level (FKGL), Simple Measure of Gobbledygook (SMOG), and Gunning Fog Index (GFOG), were relatively higher for the dimensions of etiology, risk factors, and genetic-related issues and diagnostic pathways and differential diagnosis, whereas they were relatively lower for clinical manifestations and complications and basic concepts and epidemiology. By contrast, the Flesch Reading Ease Score (FRES) showed limited variation across dimensions, suggesting that thematic differences were reflected primarily in reading burden rather than in perceived ease of reading *per se*.

Cross-platform comparisons revealed more pronounced differences in readability. ChatGPT 5.2-Thinking and DeepSeek-V3.2 exhibited more outwardly expanded profiles on ARI, FKGL, SMOG, and GFOG, indicating outputs at a relatively higher grade level. KIMI-K2.5 Thinking showed a comparatively higher Coleman-Liau Index (CLI). In contrast, ERNIE Bot 5.0 Preview and Tencent Yuanbao demonstrated higher FRES values and relative convergence across multiple grade-level indices, yielding outputs overall closer to a more readable style. These findings suggest a clear trade-off among models between information density and readability.

The radar chart summarizes seven readability indices, including the Automated Readability Index, Coleman-Liau Index, Flesch-Kincaid Grade Level, Flesch Reading Ease Score, Gunning Fog Index, Linsear Write Formula, and Simple Measure of Gobbledygook. The profiles compare the relative readability patterns of AI-generated texts across LLM platforms and thematic content dimensions. For ARI, CLI, FKGL, GFOG, Linsear Write Formula, and SMOG, higher values indicate greater reading difficulty or a higher estimated educational level required for comprehension. In contrast, for FRES, higher values indicate easier readability. This figure demonstrates differences in reading burden, linguistic complexity, and patient-oriented accessibility across models and topic categories. ARI, Automated Readability Index; CLI, Coleman-Liau Index; FKGL, Flesch-Kincaid Grade Level; FRES, Flesch Reading Ease Score; GFOG, Gunning Fog Index; SMOG, Simple Measure of Gobbledygook.

### Correlation analysis

**Figure 4:**
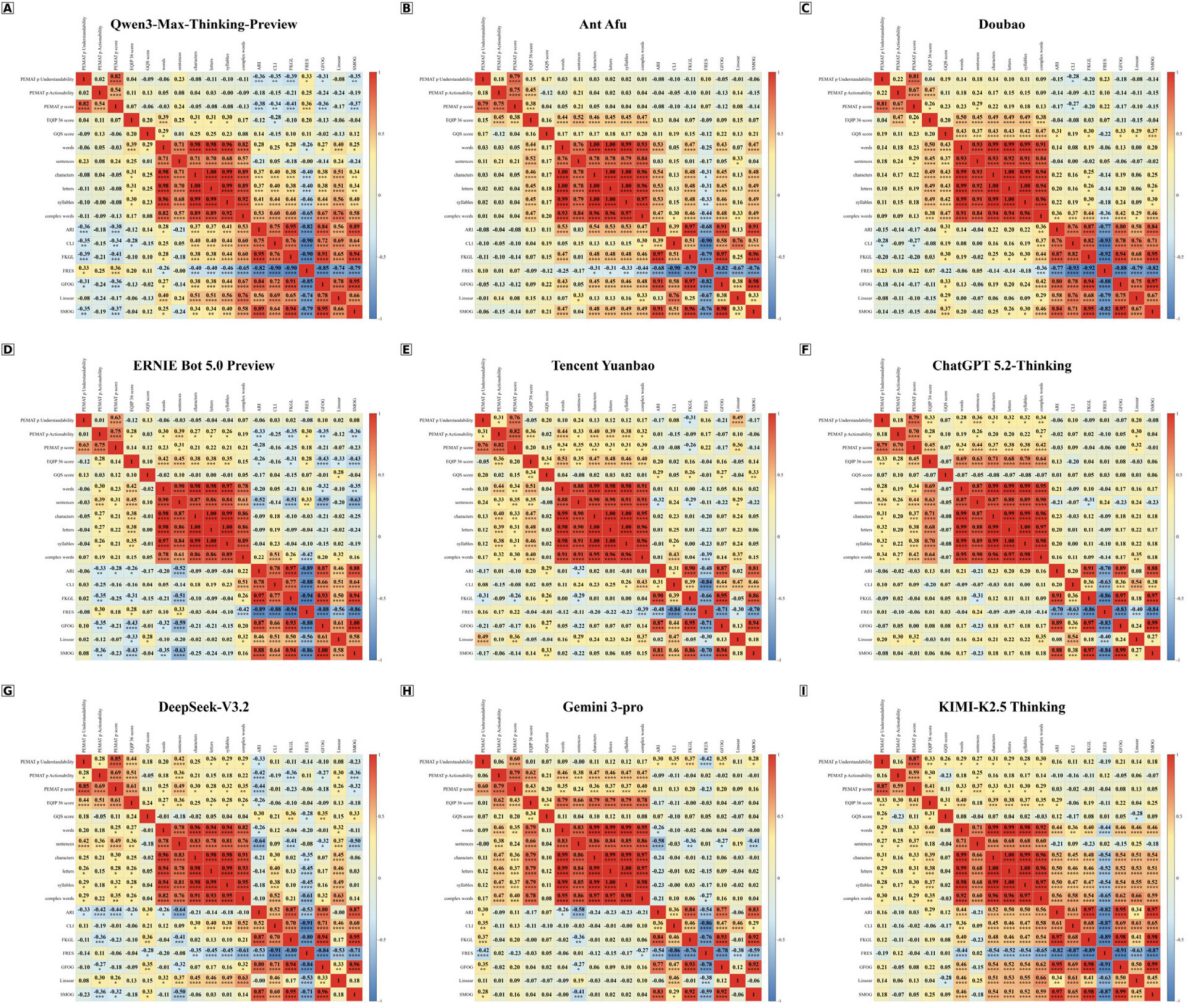
Within-platform spearman correlation heatmaps of quality, readability, actionability, and text-length indicators in LLM-generated educational texts on cardiac myxoma.

Within-platform Spearman correlation heatmaps (Figures 4A–I) showed that all models consistently formed two stable correlation modules. The first was a text length and complexity module, in which word count, sentence count, character count, letter count, syllable count, and complex word count were all strongly positively correlated. The second was a grade-level readability module, characterized by strong correlations among the Automated Readability Index (ARI), Flesch-Kincaid Grade Level (FKGL), Gunning Fog Index (GFOG), Simple Measure of Gobbledygook (SMOG), and Coleman-Liau Index (CLI), all of which were negatively correlated with the Flesch Reading Ease Score (FRES). These findings suggest that reading burden was driven primarily by lexical and syntactic complexity.

Among the quality metrics, the understandability and composite scores of PEMAT-P were strongly positively correlated across all platforms (r = 0.63–0.85). Actionability was also moderately to strongly correlated with the PEMAT-P composite score, although the degree of coupling varied across platforms. In Doubao and Ant Afu, understandability and actionability were strongly correlated (r = 0.75–0.86), whereas in Gemini they were nearly independent (r = 0.06). EQIP-36 showed moderate to strong correlations with text length indicators overall, particularly in Gemini, ChatGPT 5.2-Thinking, and DeepSeek-V3.2, suggesting that information completeness on some platforms depended more heavily on output length. By contrast, the Global Quality Score (GQS) showed weak or near-zero correlations with most variables, indicating that subjective overall quality ratings were not closely aligned with structured quality or readability metrics.

The direction of the correlations between quality and readability also differed across platforms. In Qwen3-Max-Thinking-Preview and DeepSeek, grade-level readability indices were more clearly negatively correlated with PEMAT-P scores, especially understandability, suggesting that greater reading burden might impair comprehension. In ERNIE Bot 5.0 Preview and Tencent Yuanbao, the negative correlations between text length and grade-level indices were more pronounced, indicating that longer texts did not necessarily impose a higher reading burden.This pattern suggests that different models adopt different trade-offs among information density, readability, and usability.

**Figure 5:**
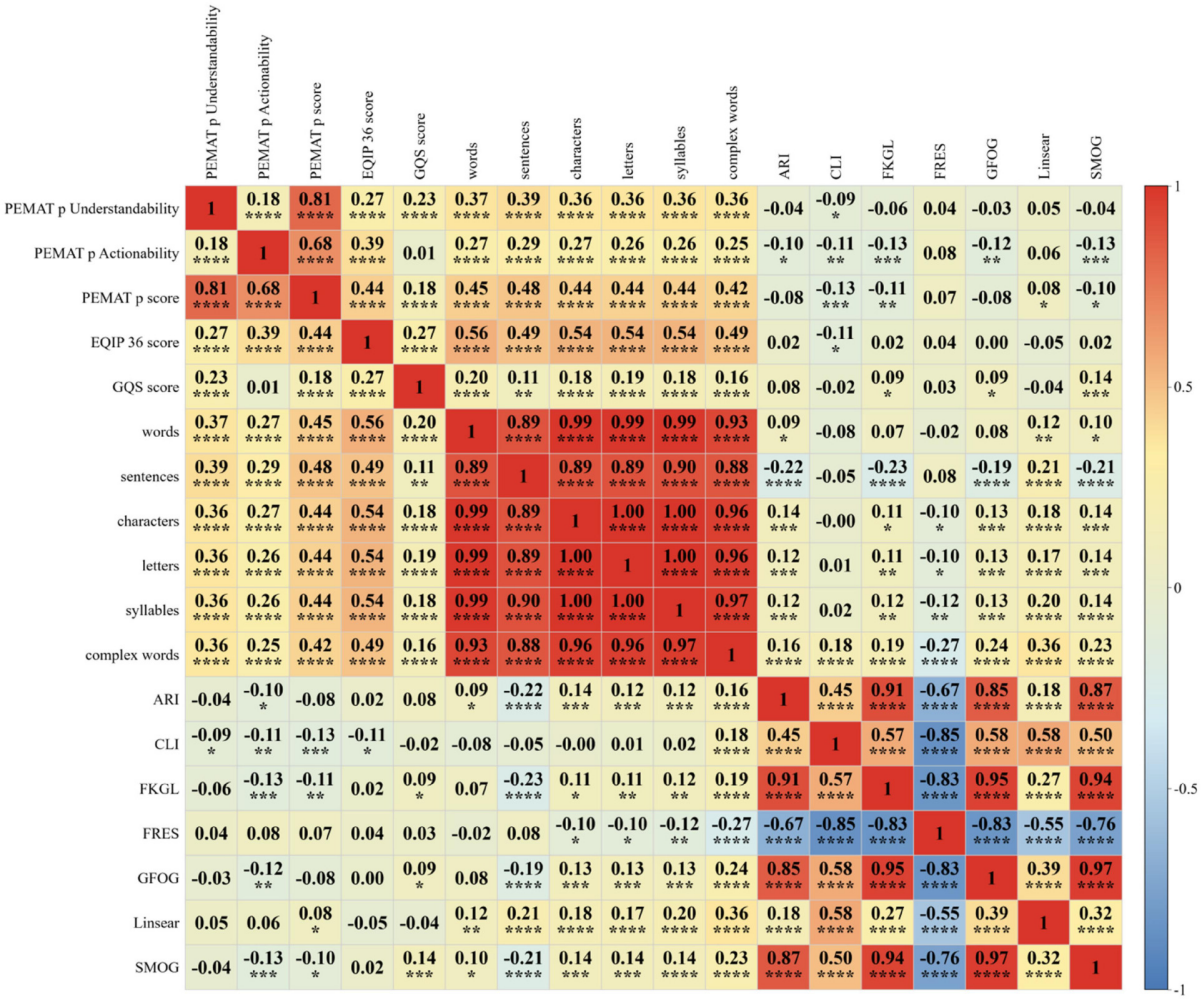
Pooled cross-platform correlations among quality, readability, actionability, and text-length indicators in LLM-generated educational texts on cardiac myxoma.

The overall correlation heatmap (Figure 5) further showed that the strongest association was observed between PEMAT-P understandability and the PEMAT-P composite score (r = 0.81). Actionability was also strongly positively correlated with the composite score (r = 0.68), and both were moderately positively correlated with EQIP-36 (r = 0.27–0.44). GQS was only weakly correlated with PEMAT-P and EQIP-36 and was almost uncorrelated with actionability. Text length indicators were highly intercorrelated, as were the grade-level readability indices, all of which were significantly negatively correlated with FRES. Quality metrics showed moderate positive correlations with text length and complexity, but only weak correlations with grade-level readability indices, suggesting that improvements in quality were more likely to accompany increases in text length and information density rather than marked reductions in reading difficulty.

Panels A–I present separate Spearman correlation matrices for the nine LLM platforms, with 60 responses analyzed for each platform. The variables include PEMAT-P understandability, PEMAT-P actionability, PEMAT-P composite score, EQIP-36, GQS, word count, sentence count, character count, letter count, syllable count, complex word count, and seven readability indices. Color intensity represents the strength of the correlation coefficient, whereas color direction indicates positive or negative associations. These heatmaps show platform-specific relationships among information quality, understandability, actionability, linguistic complexity, text length, and reading burden.

The heatmap presents the overall Spearman correlation matrix based on all 540 AI-generated texts from the nine LLM platforms. Variables include quality scores, actionability scores, readability indices, and text-length indicators. Positive correlations indicate that two variables tend to increase together, whereas negative correlations indicate inverse relationships. This pooled analysis was used to identify global association patterns before conducting partial correlation network analysis. The figure highlights the clustering of text-length indicators, the clustering of grade-level readability indices, and the relationships between patient education quality metrics and reading burden.

### Network analysis

#### Network structure

**Figure 6:**
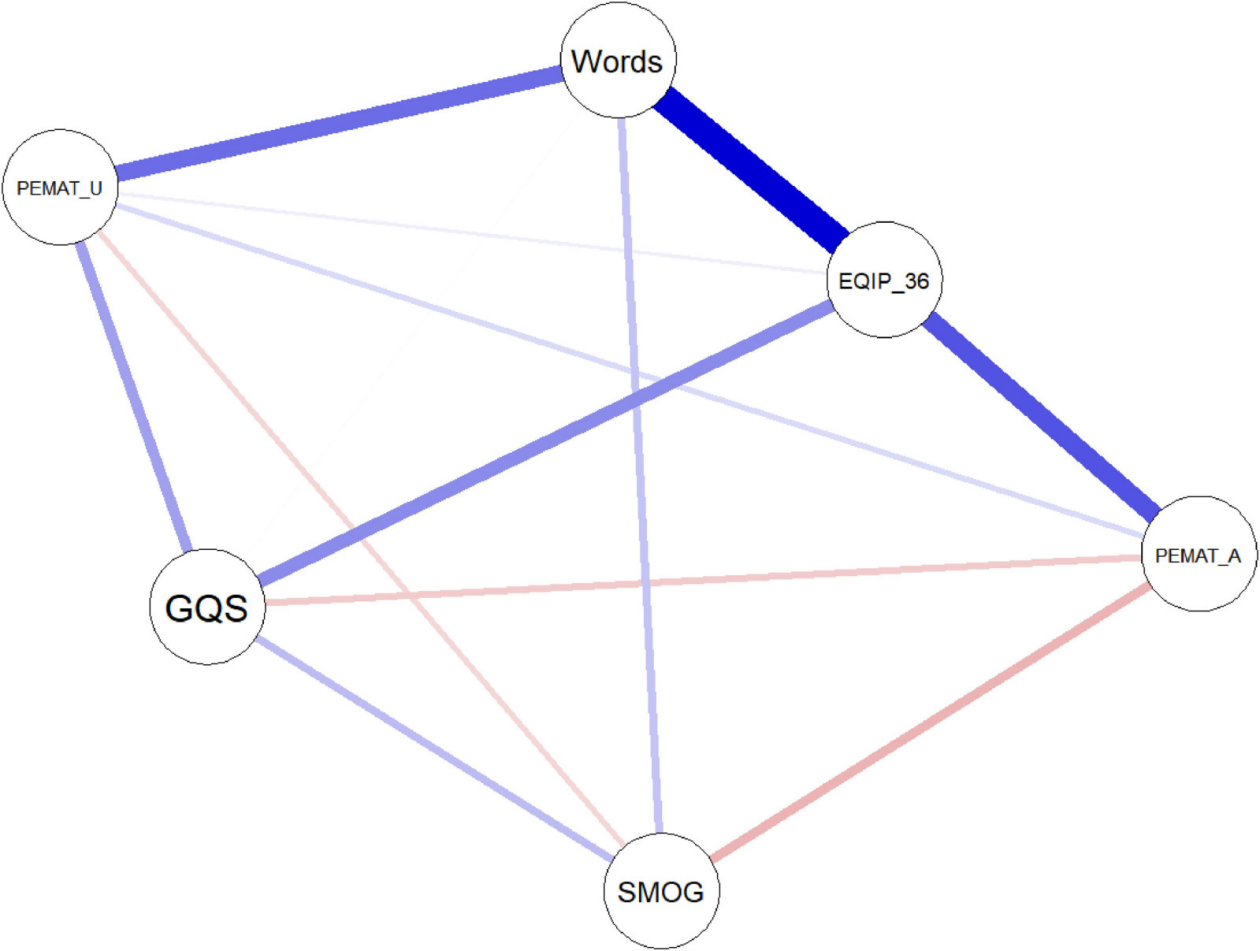
Partial correlation network of quality, readability, and actionability in LLM-generated educational texts on cardiac myxoma.

Based on the extended Bayesian information criterion graphical least absolute shrinkage and selection operator (EBICglasso) partial correlation network estimated from data after nonparanormal (NPN) transformation, the overall network showed a sparse structure, suggesting that the independent associations among quality, readability, and actionability in cross-platform LLM-generated texts were primarily driven by a limited number of key pathways (Figure 6). Edge thickness represents the strength of the association, whereas edge color indicates its direction.

In terms of network structure, the Words–EQIP-36 edge showed the strongest positive partial correlation and served as the principal backbone connection. Meanwhile, EQIP-36 maintained stable positive connections with the PEMAT-P actionability score and the Global Quality Score (GQS), suggesting coordinated variation among structural/information quality, subjective overall quality assessment, and actionability. In contrast, the Simple Measure of Gobbledygook (SMOG) index showed negative connections with PEMAT-P–related indicators, suggesting that greater readability difficulty may weaken text actionability and understandability. Overall, EQIP-36 and Words were located closer to hub positions in the network, reflecting their potential driving roles in the interrelationships among multidimensional indicators.

The network was estimated using a regularized Gaussian graphical model with the EBICglasso method after nonparanormal transformation. Nodes represent six continuous indicators: PEMAT-P understandability, PEMAT-P actionability, EQIP-36, GQS, SMOG, and word count. Edges represent regularized partial correlations after controlling for all other variables in the network. Thicker edges indicate stronger conditional associations. Positive edges indicate positive partial correlations, whereas negative edges indicate negative partial correlations. The network illustrates the conditional association structure among information quality, actionability, readability burden, and text length. EBICglasso, extended Bayesian information criterion graphical least absolute shrinkage and selection operator; PEMAT-P, Patient Education Materials Assessment Tool for Printable Materials; EQIP-36, Ensuring Quality Information for Patients; GQS, Global Quality Score; SMOG, Simple Measure of Gobbledygook.

#### Network centrality analysis

**Figure 7:**
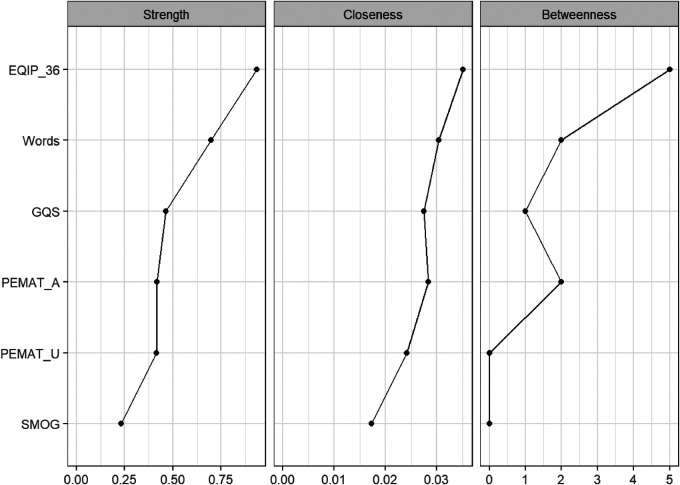
Centrality indices of nodes in the partial correlation network.

After further evaluating node centrality in the Gaussian graphical model (GGM) partial correlation network estimated using EBICglasso, the Strength results indicated that network influence was primarily concentrated in metrics related to structural quality and text length (Figure 7). Specifically, EQIP-36 showed the highest Strength, followed by Words, suggesting that these two indicators had greater total direct connection strength with other dimensions and may occupy key hub positions in the quality-readability-actionability interplay of cross-platform LLM-generated texts. GQS and PEMAT-P actionability/understandability scores showed intermediate Strength values, indicating that although overall quality assessment and actionability/understandability participated in the network structure, their overall influence was weaker than that of EQIP-36 and Words. SMOG showed the lowest Strength, suggesting that, after controlling for other variables, its independent associations with the remaining network indicators were relatively limited. Given that the stability of Closeness and Betweenness is relatively constrained in small networks, the findings of this study were interpreted primarily on the basis of Strength as the principal centrality metric.

The figure shows the centrality estimates of the six nodes included in the regularized partial correlation network. Strength was used as the primary centrality metric because it reflects the total absolute connection strength between a given node and all other nodes in the network. Higher Strength values indicate that the corresponding variable occupies a more central position in the network structure. The centrality results were used to identify key indicators involved in the interaction among information quality, text length, readability burden, and actionability. PEMAT-P, Patient Education Materials Assessment Tool for Printable Materials; EQIP-36, Ensuring Quality Information for Patients; GQS, Global Quality Score; SMOG, Simple Measure of Gobbledygook.

#### Stability analysis

**Figure 8:**
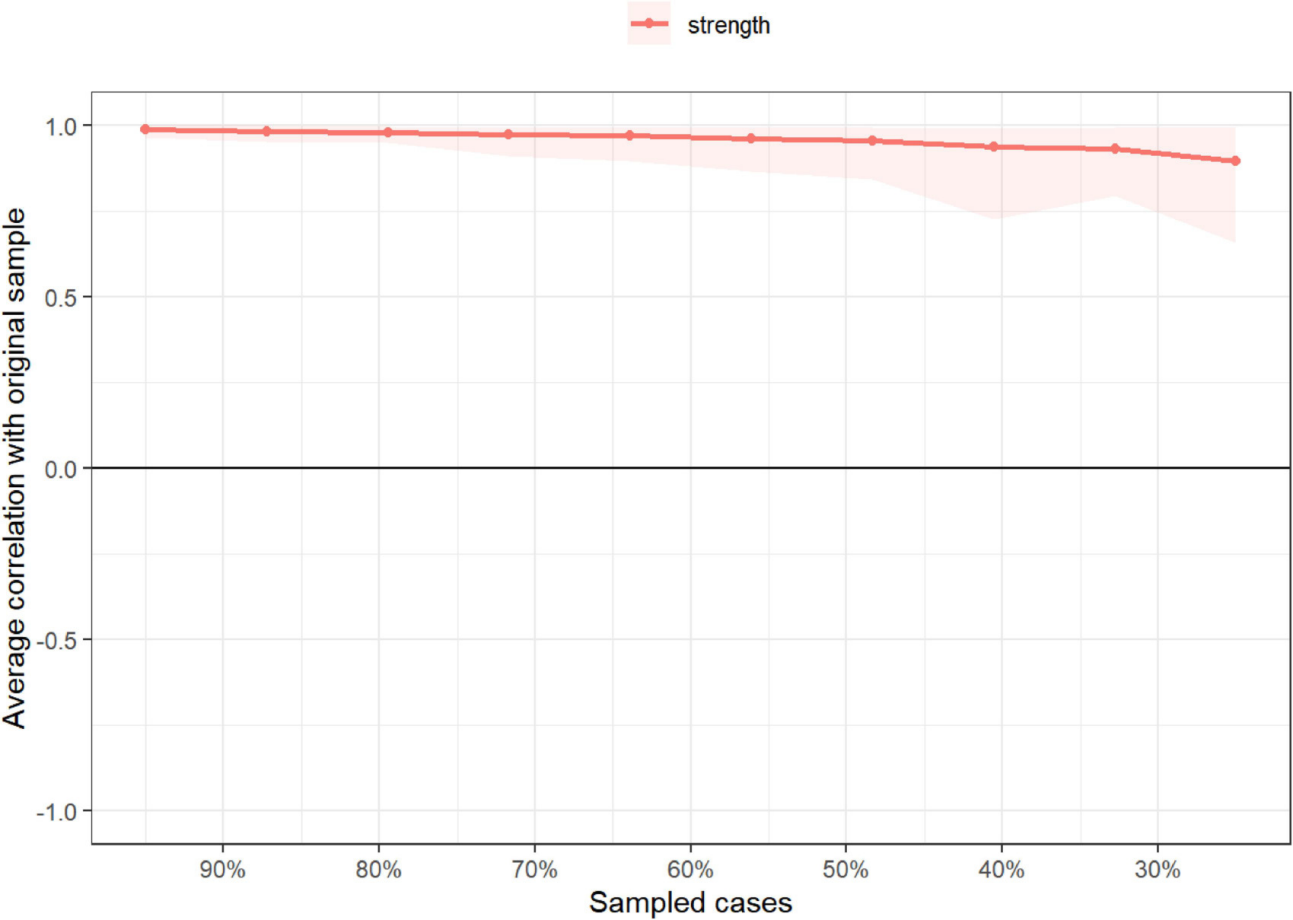
Stability analysis of the partial correlation network based on case-dropping bootstrap.

To assess the robustness of network estimation, we used a case-dropping bootstrap procedure to examine the stability of edge weights and centrality indices (Figure 8). The results showed that the correlation stability coefficient for edge weights (CS_edge) was 0.75, reaching the highest level tested. In at least 95% of bootstrap resamples, edge weights remained correlated with those from the original sample at r ≥ 0.70 even when up to 75% of cases were dropped. Meanwhile, the correlation stability coefficient for Strength centrality (CS_strength) was 0.672, indicating that after the removal of up to approximately 67.2% of the sample, the ranking of Strength centrality still maintained a correlation of r ≥ 0.70 with the original results in 95% of resamples.

The stability curves further showed that, as the retained sample proportion decreased (ie, as the proportion of dropped cases increased), the mean correlation between Strength centrality and the original sample declined slightly but remained at a relatively high level overall. The confidence bands widened modestly at higher case-dropping proportions, suggesting greater uncertainty under extreme subsampling conditions. Overall, the network showed good to strong stability in both edge-weight estimation and Strength centrality (CS > 0.5), supporting the interpretation and reporting of the major edges and key nodes.

The figure shows the robustness of network edge weights and centrality estimates under repeated case-dropping bootstrap resampling. The case-dropping procedure evaluates whether the estimated network structure remains stable when increasing proportions of cases are removed from the dataset. The correlation stability coefficient indicates the maximum proportion of cases that can be dropped while maintaining a correlation of at least 0.70 with the original estimates in 95% of bootstrap samples. Higher stability coefficients support the reliability of the major network edges and Strength centrality interpretation.

### Latent profile analysis (LPA)

#### Model fit and class enumeration

One- through five-class latent profile models were compared (Table 6). As the number of classes increased, the log-likelihood (LogLik) continuously improved, whereas the Akaike information criterion (AIC) and sample-size adjusted Bayesian information criterion (SABIC) generally decreased. In addition, the bootstrap likelihood ratio test (BLRT) was significant for the two- through five-class models (all *P* = 0.010), indicating better fit than the corresponding model with one fewer class. After jointly considering the Bayesian information criterion (BIC), entropy, and class size, the three-class model was identified as the optimal solution. This model had the lowest BIC (10001.05), an entropy of 0.778, and a minimum class proportion of 15.0%. Although the AIC decreased further in the four- and five-class models, the BIC increased, and the smallest class proportions declined to 8.7% and 8.0%, respectively, suggesting that adding further classes might lead to overextraction and reduced classification stability.

**Table 6:** Fit indices for latent profile analysis models.

Classes	LogLik	AIC	BIC	SABIC	Entropy	Smallest class proportion	BLRT *P* value
1	−4,859.636	9,831.273	10,071.6	9,893.837	1	1	—
2	−4,811.874	9,751.748	10,026.41	9,823.249	0.842	0.169	0.01
3	−4,774.026	9,692.052	10,001.05	9,772.491	0.778	0.15	0.01
4	−4,753.186	9,666.371	10,009.7	9,755.748	0.765	0.087	0.01
5	−4,736.049	9,648.098	10,025.76	9,746.413	0.745	0.08	0.01

#### Definition of phenotypic profiles of the latent classes

**Figure 9:**
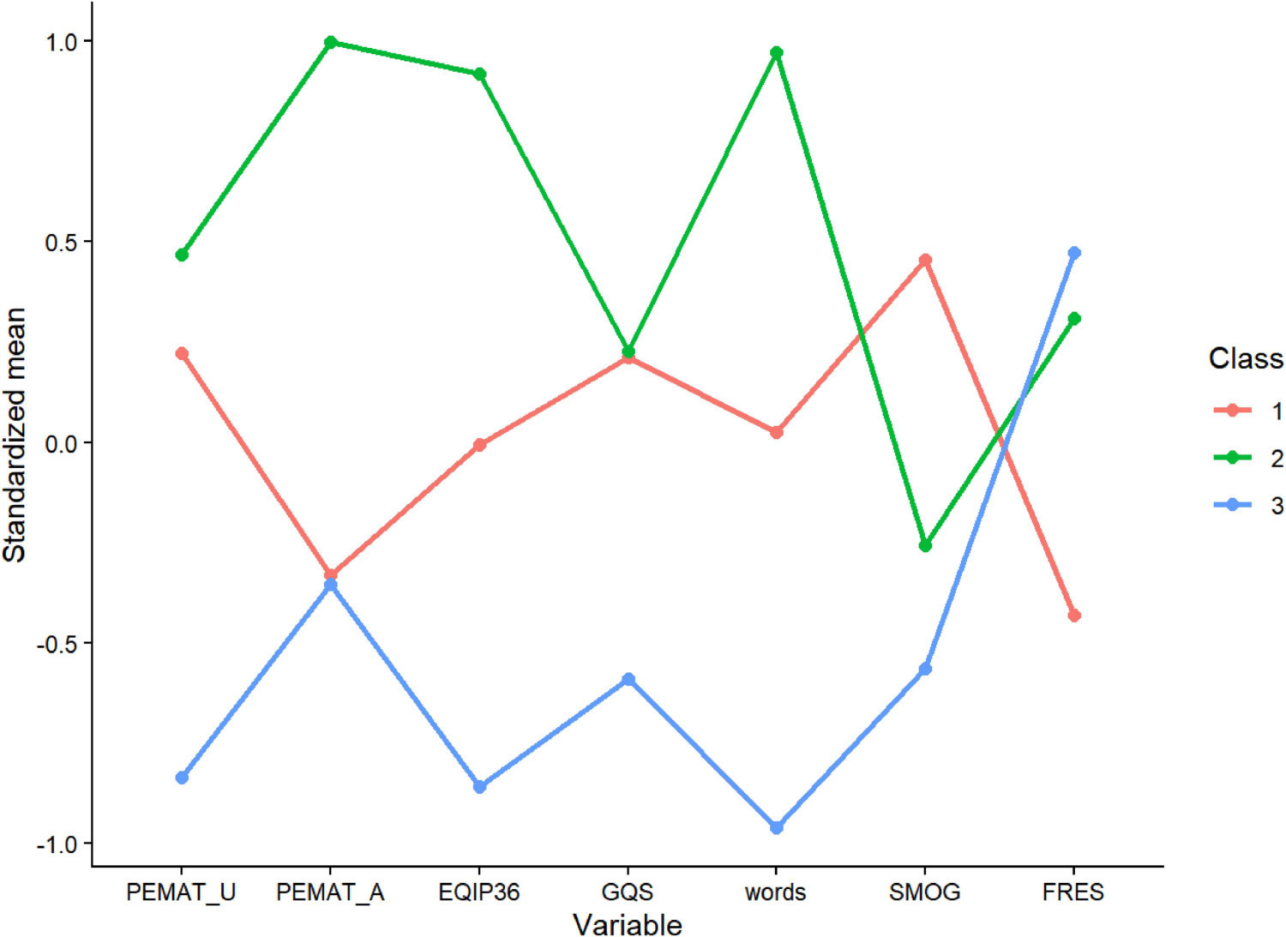
Latent profile plot of multidimensional text phenotypes identified by latent profile analysis.

Based on the optimal three-class solution, each latent class exhibited clear and discriminative phenotypic profiles (Figure 9). Class 1 showed moderate or slightly above-average levels of the PEMAT-P understandability score, Global Quality Score (GQS), and EQIP-36, but a relatively low PEMAT-P actionability score. At the same time, it had the highest Simple Measure of Gobbledygook (SMOG) index and the lowest Flesch Reading Ease Score (FRES), suggesting that although the informational quality of these texts was acceptable, their actionability was limited and their reading difficulty was relatively high. This class was therefore defined as the “moderate-quality, low-readability” type.

Class 2 had substantially above-average levels of PEMAT-P understandability, PEMAT-P actionability, and EQIP-36, as well as the highest word count. In addition, it showed a relatively low SMOG index and a relatively high FRES, indicating that these texts were more content-rich and combined higher quality, stronger understandability, and greater actionability while maintaining good readability. This class was therefore defined as the “high-quality, high-actionability” type.

Class 3 showed below-average levels across PEMAT-P understandability, PEMAT-P actionability, EQIP-36, GQS, and word count, but had the lowest SMOG index and the highest FRES. These findings suggest that although the texts in this class were shorter and easier to read, they were limited in informational quality and practical usefulness. This class was therefore defined as the “low-quality, simplified and easy-to-read” type.

The figure presents standardized mean profiles for the three latent classes identified from quality, readability, actionability, and text-length indicators. Class 1 represents the moderate-quality, low-readability phenotype, characterized by acceptable information quality but relatively high reading burden and limited actionability. Class 2 represents the high-quality, high-actionability phenotype, characterized by higher understandability, actionability, information quality, and relatively favorable readability. Class 3 represents the low-quality, simplified and easy-to-read phenotype, characterized by shorter and easier-to-read texts but lower information quality and practical usefulness. Higher standardized values indicate above-average levels of the corresponding indicator. This figure demonstrates heterogeneity in LLM-generated educational texts beyond single-platform or single-metric comparisons.

## Discussion

### Overview of the main findings

Although cardiac myxoma is a benign primary cardiac tumor, patients remain at risk for postoperative recurrence, embolic events, and heart failure. Accordingly, long-term management depends on patients' early recognition of complications and warning symptoms. In this study, we systematically evaluated the performance of nine mainstream large language models (LLMs) in generating patient education texts on cardiac myxoma. The results demonstrated substantial inter-model differences in information quality, understandability, actionability, and objective readability. ERNIE Bot 5.0 Preview and Gemini 3-pro performed relatively well in information organization and overall quality, whereas Qwen3-Max-Thinking-Preview showed weaker output stability and lower overall quality. Partial correlation network analysis further indicated a central association between text length and structural quality, while latent profile analysis identified three text phenotypes with differing textual characteristics and differential suitability for further review as draft educational materials.

### Comparison with previous studies and remaining challenges

Previous studies have evaluated the quality, readability, and actionability of LLM-generated patient education materials in several clinical contexts, including chronic heart failure, diabetes, aortic stenosis, fibromyalgia, and prostate cancer (11, 21, 22, 42–44). In addition, broader reviews have suggested that LLMs are increasingly being explored for medical communication, patient education, and oncology-related information support, but their outputs remain constrained by readability burden, incomplete action-oriented guidance, and uncertainty regarding clinical reliability (45, 46). Overall, the findings of the present study are consistent with this body of literature. In our cardiac myxoma dataset, LLM-generated texts showed substantial heterogeneity across platforms, and many outputs remained difficult to read and insufficiently actionable for direct patient-facing education. These findings further support previous evidence that structurally complete AI-generated medical information does not necessarily translate into patient-centered, understandable, or behaviorally useful educational material (11, 42–45).

However, compared with previous studies focusing primarily on common chronic diseases, common cancers, or single-system conditions (11, 21, 22, 43, 44), cardiac myxoma poses several additional educational challenges. First, cardiac myxoma is a rare primary cardiac tumor, and patients may have limited prior knowledge of the disease before diagnosis. Second, although the tumor is histologically benign, its clinical consequences may be functionally malignant because of the risks of systemic embolism, intracardiac obstruction, heart failure, syncope, stroke, and sudden death. Third, patient education in this context requires integration of cardiovascular emergency recognition, tumor-related prognosis, perioperative management, recurrence surveillance, genetic or syndrome-related risk, and long-term follow-up. Therefore, educational materials for cardiac myxoma should not only explain disease mechanisms, diagnosis, and treatment, but also provide clear behavioral instructions regarding warning symptoms, when to seek urgent care, how to participate in postoperative surveillance, and how to communicate recurrence-related concerns with clinicians.

Compared with existing literature, the present study also has several methodological extensions. Many prior studies mainly relied on descriptive comparisons of readability, quality, or accuracy scores in one or two LLMs or in a limited number of clinical questions (21, 22, 42, 43). By contrast, this study included nine LLM platforms and further applied partial correlation network analysis and latent profile analysis. These approaches allowed us to examine how information quality, readability burden, actionability, and text length co-occurred within AI-generated patient education texts and to identify latent text phenotypes with different suitability for further clinical review. This provides a more structured framework for screening AI-generated educational drafts before human editing. Nevertheless, these methodological extensions do not eliminate several important limitations. Similar to many previous studies, we evaluated textual characteristics rather than real-world patient comprehension, factual accuracy, hallucination risk, guideline concordance, or downstream clinical behavior (45, 46). Therefore, our findings should be interpreted as evidence for the cautious, clinician-reviewed use of LLM-generated texts as preliminary educational drafts, rather than as evidence supporting their direct unreviewed use in patient care.

### Trade-off between information quality and reading burden across platforms

A central challenge in applying generative AI to medical and health education is to balance medical accuracy with public readability. Previous studies have shown that medical texts generated by LLMs often exceed the reading level recommended for the general public by the American Medical Association, thereby creating substantial barriers to comprehension (11, 45). Consistent findings have been reported in other disease settings. In chronic heart failure and diabetes, cross-platform studies showed that LLM-generated patient education texts exhibited acceptable informational quality in some domains but remained limited by reading burden and insufficient actionability (11, 44). In the cardiovascular field, Rouhi et al. further found that although ChatGPT-3.5 and Bard improved the readability of aortic stenosis patient education materials, the rewritten texts still did not reach the recommended sixth-grade reading level (43). Our findings are consistent with this concern. Texts generated by ChatGPT 5.2-Thinking and DeepSeek-V3.2 tended to correspond to higher grade levels on ARI, FKGL, and SMOG, indicating that, in the context of cardiac myxoma, these outputs were accompanied by longer sentences and denser medical terminology.

The network centrality analysis further supports this interpretation. The strongest positive edge was observed between word count and EQIP-36, indicating that greater text length co-occurred with higher information-quality scores across platforms. However, lengthy and information-dense texts may reduce the likelihood that the target audience will derive meaningful health literacy benefits (47). Notably, some models achieved above-average quality scores while maintaining relatively good readability, suggesting that information quality and reading burden are not inherently irreconcilable. Together with prior findings in heart failure, diabetes, and aortic stenosis, our results suggest that the trade-off between informational completeness and patient-oriented readability may be a recurring limitation of current generative AI systems in health education (11, 43, 44). Future efforts to develop disease-specific educational corpora should incorporate prompt-engineering strategies tailored to cardiac oncology and related cardiovascular conditions, together with plain-language instructions, to help reduce patient reading burden while preserving medical accuracy.

### Limited clinical actionability: an educational challenge in cross-disciplinary conditions

The most prominent barrier to the practical use of these outputs as standalone patient-facing educational texts identified in this study was the uniformly low performance of all models on the actionability domain of PEMAT-P. Patients after cardiac myxoma resection remain at risk of systemic embolism and heart failure due to residual microemboli and local recurrence. Accordingly, they require specific and actionable guidance on symptom recognition and appropriate responses. However, the texts generated by current models largely remained at the level of pathology- and disease-related explanations and lacked stepwise, behavior-oriented instructions for patients. Recent studies on the use of artificial intelligence for patient education in chronic heart failure and fibromyalgia have similarly shown that model-generated materials are substantially inferior to professionally developed educational materials in providing actionable recommendations (11, 42).Importantly, PEMAT-P and EQIP-36 scores did not significantly differ across the six thematic modules, suggesting that limited actionability was not confined to technically oriented prompts alone and may reflect a broader characteristic of current LLM-generated educational texts in this setting.

The partial correlation network further highlighted a structural pattern potentially related to this deficiency. Readability difficulty indices were consistently negatively correlated with PEMAT-P actionability, suggesting that greater syntactic complexity may be linked to increased comprehension burden and lower textual actionability. One possible explanation is that general-purpose LLMs may have difficulty translating abstract medical knowledge into concrete self-management instructions for patients within the educational dimensions assessed in this study. At the same time, this interpretation should be made cautiously, because the present study evaluated textual actionability rather than actual patient behavior, comprehension, or downstream clinical responses.

### Identification of latent text phenotypes and precision health information delivery

Latent profile analysis (LPA) is a person-centered statistical approach that has been widely used to identify group heterogeneity in health literacy and engagement among patients with chronic diseases (19). In the present study, we extended this method to the evaluation of medical artificial intelligence outputs and identified three text phenotypes: the moderate-quality, low-readability type, the high-quality, high-actionability type, and the low-quality, simplified and easy-to-read type.

These findings move beyond the limitations of prior studies that relied solely on single-dimension mean scores and provide a more accurate representation of the output quality structure arising from the joint effects of multiple indicators. Our analysis further showed that the high-quality, high-actionability phenotype accounted for only a limited proportion of texts, yet most closely approximated the standard of more suitable patient-facing educational drafting within the dimensions assessed in this study. Rather than supporting direct implementation without review, this finding suggests that phenotype-based evaluation may help clinicians and researchers identify which AI-generated texts appear more suitable for further human editing and review. In this context, phenotype-informed screening may be useful as a research or quality-control framework for stratifying draft educational materials, especially when considering audiences with differing levels of cognitive and health literacy capacity.

### Clinical implications, limitations, and future directions

This study provides evidence to support the more standardized evaluation of generative AI in specialized education on complex cardiac tumors. When using such tools to assist in drafting patient education materials, medical professionals should still conduct rigorous human review, with particular attention to supplementing procedural instructions and action-oriented information. Model developers, in turn, should incorporate authoritative oncology and cardiovascular clinical guidelines into domain-specific fine-tuning to reduce the current tendency toward relying heavily on text-length expansion alone (46).

This study also has several limitations. First, because a cross-sectional design was used, we were unable to assess long-term performance changes associated with the continuous iteration of LLMs. Second, although the question set was expert-curated to support standardized cross-platform benchmarking, some prompts may not fully reflect the wording, priorities, and urgency-focused concerns of real-world patients. Third, to control baseline variables, all tests were conducted using English-language prompts; future studies should verify whether these findings remain consistent across multilingual and cross-cultural settings. Fourth, only text-based outputs were evaluated, and multimodal content, such as images and videos, which may improve the efficiency of physician-patient communication, was not included. Fifth, Bonferroni correction was applied conservatively in the *post hoc* multiple-comparison procedures and may therefore have reduced statistical power for detecting some smaller between-platform or between-dimension differences. In addition, although we assessed text quality, readability, understandability, and actionability using validated instruments, we did not directly evaluate factual accuracy, omission of key information, hallucination risk, appropriateness of recommendations, concordance with clinical guidelines, or real-world patient comprehension. Therefore, the present findings should be interpreted as a comparative assessment of textual characteristics of LLM-generated educational materials, rather than as a direct evaluation of clinical safety or effectiveness.

## Conclusion

Within an expert-curated educational question set spanning the disease course of cardiac myxoma, this study systematically characterized the multidimensional features and key limitations of patient education texts generated by current mainstream LLMs. The findings demonstrated substantial heterogeneity across AI platforms in information quality, objective readability, and clinical actionability. Although some models were capable of producing structurally complete, professionally styled medical texts, their overall suitability as standalone patient-facing materials appeared to be commonly constrained by an excessively high cognitive reading burden and a marked lack of practical behavioral guidance.

Partial correlation network analysis and latent profile analysis provided additional structural insights into how these evaluated text features co-occurred within this dataset. Specifically, higher word count was closely associated with better information-quality scores, whereas complex syntactic structures were associated with lower textual actionability. Among the three latent text phenotypes identified, only a very small proportion of outputs were able to simultaneously achieve high quality and high actionability, falling well short of the desired standards for patient education in clinical oncology and chronic disease management.

Therefore, the implementation of generative AI in specialty nursing and the long-term management of patients with tumors should not be based uncritically on the generic output of any single platform. Health care institutions may consider establishing automated prescreening and routing mechanisms based on text phenotypes to enable tailored health communication for populations with different levels of cognitive and health literacy. At the same time, because this study did not directly assess factual accuracy, guideline concordance, or patient safety, these findings are best understood as evidence for the careful, clinician-reviewed use of LLM-generated texts as draft educational materials rather than as support for direct unreviewed deployment in patient care. Future model optimization should incorporate alignment with authoritative medical guidelines, move beyond a text-length-dependent generation strategy, and place greater emphasis on concrete disease-response instructions and plain-language expression if LLM-generated texts are to be used more appropriately in patient education workflows. Such efforts may help improve the appropriateness of generative AI as an adjunctive tool for clinician-patient communication.

## Data Availability

The original contributions presented in the study are included in the article/Supplementary Material, further inquiries can be directed to the corresponding author/s.
